# miR156- and miR171-Binding Sites in the Protein-Coding Sequences of Several Plant Genes

**DOI:** 10.1155/2013/307145

**Published:** 2013-07-11

**Authors:** Assyl Bari, Saltanat Orazova, Anatoliy Ivashchenko

**Affiliations:** Al-Farabi Kazakh National University, 71 Al-Farabi Avenue, Building No.6, Almaty 050038, Kazakhstan

## Abstract

We identified the interaction sites of several miRNAs with the mRNAs from paralogs and orthologs of the *SPL* and *HAM* genes in *A. thaliana*. miRNAs from the miR156 and miR157 families in *A. thaliana* are shown to have binding sites within the mRNAs of *SPL* genes. The ath-miR156a–j binding sites located in the mRNAs of the *SPL* paralogs contain the sequence GUGCUCUCUCUCUUCUGUCA. This sequence encodes the ALSLLS motif. miR157a–d bind to mRNAs of the *SPL* family at the same site. We suggest merging the miR156 and miR157 families into one family. Several *SPL* genes in eight plants contain conserved miR156 binding sites. GUGCUCUCUCUCUUCUGUCA polynucleotide is homologous in its binding sites. The ALSLLS hexapeptide is also conserved in the SPL proteins from these plants. Binding sites for ath-miR171a–c and ath-miR170 in *HAM1*, *HAM2*, and *HAM3* paralog mRNAs are located in the CDSs. The conserved miRNA binding sequence GAUAUUGGCGCGGCUCAAUCA encodes the ILARLN hexapeptide. Nucleotides within the *HAM1*, *HAM2*, and *HAM3* miRNA binding sites are conserved in the mRNAs of 37 orthologs from 13 plants. The miR171- and miR170-binding sites within the ortholog mRNAs were conserved and encode the ILARLN motif. We suggest that the ath-miR170 and ath-miR171a–c families should be in one family.

## 1. Introduction

Individual microRNAs (miRNAs) and their families can be identical or very similar in closely related and phylogenetically distant plant species [1, 2]. Therefore, it is important to determine the properties of miRNA-binding sites in the protein-coding sequences (CDSs) of paralogous and orthologous genes. In plants, miRNAs regulate the expression of many genes involved in plant morphogenesis and development [3–7] and resistance to biotic and abiotic stresses [2, 8–10]. The number of identified plant miRNAs is growing, and the main challenge is to clearly identify their targets. Many miRNA-binding site prediction programs such as miRanda (http://www.microrna.org/microrna/getMirnaForm.do) [11], DIANA microT (http://diana.cslab.ece.ntua.gr/DianaTools/index.php?r=microtv4/index) [12], and PicTar (http://pictar.mdc-berlin.de/) [13] search for the sites in the 3′-untranslated region (3′UTR) of the mRNA. However, in plant and animal cells, miRNA-binding sites have been identified in the 5′-untranslated region (5′UTR) and CDS [14–18]. Computational methods can predict many miRNA-binding sites in mRNAs. However, a significant proportion of false-positive miRNA-binding sites are identified. Therefore, it is necessary to develop methods of improving the reliability of site prediction. One way to improve the reliability of binding site prediction is to check if the sites are present in orthologous genes. The aim of our research is to identify the interaction sites of several miRNAs within the CDSs of paralogous and orthologous mRNAs and establish the features of these interactions. *SPL* and *HAM* genes code for transcription factors and play a key role in the regulation of plant reproductive development [19, 20]. The expression of these genes is controlled by miRNAs. In this paper, we present the characteristics of the binding sites for the miR156, miR157, miR170, and miR171 families in several paralogous and orthologous *SPL* and *HAM* genes in *A. thaliana* and other plant species.

## 2. Materials and Methods

The gene sequences from *Arabidopsis lyrata, Arabidopsis thaliana, Brachypodium distachyon, Glycine max, Medicago truncatula, Oryza sativa, Physcomitrella patens, Populus trichocarpa, Ricinus communis, Selaginella moellendorffii, Sorghum bicolor, Vitis vinifera, *and* Zea mays* were obtained from GenBank (http://www.ncbi.nlm.nih.gov/). The miRNAs sequences were retrieved from miRBase (http://www.mirbase.org/). The free energy (Δ*G*) of hybridization between miRNAs and mRNAs, the position of potential binding sites, and the interaction schemes were calculated using the RNAHybrid 2.1 program (http://bibiserv.techfak.uni-bielefeld.de/rnahybrid/) [21]. The E-RNAhybrid program (http://sites.google.com/site/malaheenee/software/) was used to compute the Δ*G*/Δ*G*_*m*_ value and *P* value. The Δ*G*/Δ*G*_*m*_ value was used as a comparative criterion for the miRNA and mRNA interaction force. A Δ*G*/Δ*G*_*m*_ value of more than 75% indicates a significant degree of complementarity between the miRNA and its target. This percentage corresponds to *P* < 0.005. The Δ*G* value and its standard deviation were used to determine the validity of predicted miRNA-binding sites in the mRNA. The maximal interaction energy (Δ*G*_*m*_) for miR156, miR157, miR170, miR171, and their families was equal to the binding energy of perfectly complementary sequences. Graphs of the nucleotide and amino acid sequence variability were created by using the WebLogo program (http://weblogo.berkeley.edu/) [22]. To improve the reliability of predicted miRNA-binding sites in the mRNAs of genes in *A*. *thaliana*, we confirmed their presence in the mRNAs of orthologous genes in other plants.

## 3. Results

### 3.1. Binding of the miR156 and miR157 Families with the mRNAs of *SPL* Paralogs

We found that, among 328 miRNAs in *A. thaliana,* the miR156 and miR157 families are shown to have strong binding sites within the squamosa promoter binding protein-like (*SPL*) gene family of transcription factors. The miR156 family consists of 10 miRNAs (miR156a–j), and miR156a–f have identical nucleotide sequences (miRBase). miR156 family members are predicted to be associated with the mRNAs of genes encoding the DNA-binding proteins SPL1–SPL16 with varying degrees of prediction reliability. Among the 16 genes in this family, CDSs of eight paralogs have been targeted by miR156a: *AT1G27360 (SPL11), AT1G27370 (SPL10), AT1G69170 (SPL6), AT2G42200 (SPL9), AT3G57920 (SPL15), AT5G43270 (SPL2), AT5G50570 (SPL13)*, and *AT5G50670 (SPL13)*. The genes *AT5G50570* and *AT5G50670* have significant conservation and are located at a distance of 33 kilobases (kb) from each other. Therefore, we only studied the properties of the miRNA-binding sites within the *AT5G50570* gene. The miR156a-binding sites within the *SPL* mRNAs are identical and consist of the conserved nucleotides GUGCUCUCUCUCUUCUGUCA. The open reading frame encoding the conserved ALSLLS motif begins with the GCU triplet of the miRNA-binding site sequence.

**Figure 1:**
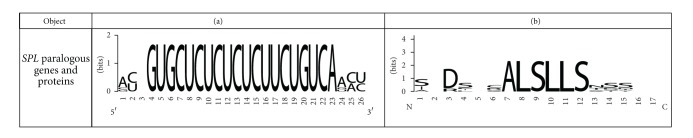
The variability of nucleotides (a) in the binding sites of miR156a with mRNA of *SPL *paralogs and the variability of amino acids (b) in SPL proteins containing the ALSLLS hexapeptide in* A. thaliana*.

Table 1 shows the nucleotide sequences of the miR156a-binding sites in the mRNAs of eight *SPL* genes and the amino acid sequences of the corresponding paralogous SPL proteins containing the ALSLLS oligopeptide. The first two nucleotides (GU) of the oligonucleotide are involved in miRNA-binding; the second and third positions are part of the nonconserved codon (NGU) of paralogous genes and, therefore, the corresponding amino acids are variable (Figure 1). This variability may indicate the importance of the GU dinucleotide in enhancing the binding of miR156a with the mRNAs.

The Δ*G*/Δ*G*_*m*_ value for the miR156a-binding sites ranged from 90.2% to 91.4%, which indicates a strong interaction between this miRNA and the mRNAs of the *SPL* gene family (Table 2). The sequences of miR156g, miR156h, miR156i, and miR156j differ from the miR156a–f sequence by one or two nucleotides; however, miR156a–j bind to the same site within each of the *SPL* paralog mRNAs, which is specific for the miR156 family. The Δ*G*/Δ*G*_*m*_ value varied from 88.4% for miR156g to 100% for miR156j (Table 2). Thus, the binding is strong in all cases.

The miR157a–c sequences differ from the miR156a sequence by one nucleotide at the 5′-end. The miR157d sequence differs by one nucleotide from the miR156h sequence and by two nucleotides from miR156a sequence. miR157a–d bind to the mRNAs of the *SPL* family at the same site as miR156a–j (Table 2). The Δ*G*/Δ*G*_*m*_ value of the miR157a–d-binding sites ranged from 89.5% to 92.5% (Table 2). Therefore, we suggest that the miRNAs of the miR156 and miR157 families belong to the same family. For example, *Oryza sativa* and *Zea mays* have only the miR156 family and not the miR157 family (miRBase).

The *SPL3 *and* SPL5 *mRNA-binding sites for miR156a are located in the 3′UTR, and their nucleotide sequences have significant conservation with those in the CDS. The Δ*G*/Δ*G*_*m*_ value for these binding sites was 93.1% and 84.7%, respectively.

Table 3 represents the interaction schemes for the miR156 and miR157 families with the mRNAs from *AT1G27360*, *AT1G27370*, *AT1G69170*, *AT2G42200*, *AT3G57920*, *AT5G43270*, and *AT5G50570* paralogs. The position of the binding sites for various miRNAs differs in paralogous genes. Thus, the binding of the miRNA with the mRNA occurs over the entire nucleotide sequence, not only within the “seed” region.

### 3.2. Binding of the miR156 Family with the mRNAs of* SPL* Orthologs

**Figure 2:**
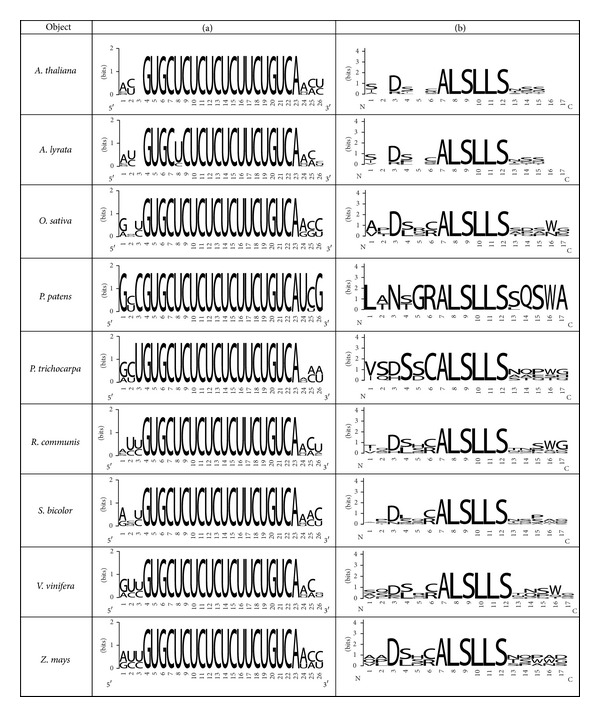
The variability of nucleotides (a) in the binding sites of miR156a with mRNAs of *SPL *orthologs and the variability of amino acids (b) in SPL proteins that contain the ALSLLS hexapeptide.

*SPL* genes from *A. lyrata, O. sativa, Populus trichocarpa, Physcomitrella patens, Ricinus communis, Sorghum bicolor, Vitis vinifera, *and *Z. mays* are targeted by the miR156 family. For all of the studied genes, the GUGCUCUCUCUCUUCUGUCA polynucleotide is completely conserved in the ath-miR156a-binding sites within the mRNA (Figure 2(a)). Consequently, the ALSLLS hexapeptide is also conserved in the SPL proteins of these plant species (Figure 2(b)). High variability of the nucleotide sequences adjacent to the binding site and therefore variability of the amino acids before and after the ALSLLS motif were observed (Figures 2(a) and 2(b)).

The mRNAs of the *SPL3 *orthologous genes in* A. lyrata* and* P. trichocarpa* have miR156a-binding sites in the 3′UTR. The nucleotide sequences of these sites differ slightly, and the Δ*G*/Δ*G*_*m*_ value was equal to 93.1% and 87.3%, respectively. The *SPL5* mRNA from *A. thaliana* and *V. vinifera* also has miR156a-binding sites in the 3′UTR. The Δ*G*/Δ*G*_*m*_ value for these sites was equal to 84.7% and 91.4%, respectively. The *SPL5 *mRNAs from* P. patens* and *Z. mays* have miR156a-interaction sites within the CDS. The Δ*G*/Δ*G*_*m*_ value for these binding sites was equal to 90.9% and 90.7%, respectively. We suggest that the location of the binding sites in the CDS and 3′UTR of *SPL3* and *SPL5* orthologs may change because of their close position to the border between the CDS and 3′UTR.

### 3.3. Binding of miR171a–c and miR170 with the mRNAs of HAM Paralogs

We have determined the binding sites for ath-miR171a within the mRNAs of *AT4G00150 (HAM3)*, *AT2G45160 (HAM1)*, and *AT3G60630 (HAM2)*. These genes belong to the GRAS transcription factor family in *A*. *thaliana* [20]. The binding sites for ath-miR171a in the CDS contain the GAUAUUGGCGCGGCUCAAUCA polynucleotide, which encodes the ILARLN hexapeptide in the corresponding proteins (Table 4).

The genes *HAM1*, *HAM2*, and *HAM3* are targets for ath-miR171b, ath-miR171c, and ath-miR170. The characteristics of the ath-miR171a–c and ath-miR170 interaction sites with the mRNAs of *HAM1, HAM2, and HAM3* paralogs are listed in Table 5. All of these miRNA-binding sites are located in the CDS and have the same position for each gene.

ath-miR171a binds perfectly with the mRNAs of *HAM* paralogs, and the Δ*G*/Δ*G*_*m*_ value was equal to 100%. ath-miR171b and c connect without the triplet at the 3′end; therefore, the Δ*G*/Δ*G*_*m*_ value was equal to 86.9%, which indicates a strong interaction with these RNAs. Although ath-miR170 belongs to another family, it strongly associates with each paralog of the *HAM* genes. The Δ*G*/Δ*G*_*m*_ value was 98.8%. Binding sites for ath-miR171a–c and ath-miR170 are in the CDS and their positions are the same in each paralog mRNA (Table 5). The conserved GAUAUUGGCGCGGCUCAAUCA oligopeptide encodes the conserved ILARLN hexapeptide in *HAM1*, *HAM2*, and *HAM3* paralogs (Table 4). A comparison of the predicted binding sites for ath-miR171a–c and ath-miR170 in *HAM1*, *HAM2*, and *HAM3* suggests that these miRNAs belong to the same family. We grouped these miRNAs into the ath-miR171 family because ath-miR171a has full complementarity with all of the sites in the mRNAs of *HAM1*, *HAM2*, and *HAM3* paralogs.

The nucleotide sequences of miR171a–c and miR170 form structures of complementary nucleotides with the binding sites within the mRNAs of *HAM* paralogs (Table 6). The position of the binding sites for various miRNAs differs in paralogous genes. This indicates a strong miRNA interaction with the mRNA. We noted that, in the center of these structures, there are eight GC pairs that make the main contribution to the interaction energy for the mRNA and miRNA pair. This is inconsistent with the concept in which the main contribution to the binding is made by “seeds” located at the 5′-end of the miRNA [23–25].

### 3.4. Binding of miR171a–c and miR170 with the mRNAs of *HAM* Orthologs

For each of the *HAM1*, *HAM2*, and *HAM3* paralogs, orthologs were found in 13 species *(A. lyrata, A. thaliana, Brachypodium distachyon, Glycine max, Medicago truncatula, O. sativa, P. patens, P. trichocarpa, R. communis, S. moellendorffii, S. bicolor, V. vinifera*, and *Z. mays*) and miR171a-binding sites were identified in these genes. The nucleotide sequences of the binding sites are highly conserved in the mRNAs of 37 orthologous genes (See Table S1 in Supplementary Material available online at http://dx.doi.org/10.1155/2013/307145). All of the nucleotides in the miR171a-binding site (GAUAUUGGCGCGGCUCAAUCA) were conserved and encode the same ILARLN motif (Supplemental Table S2). Nucleotides adjacent to the ath-miR171a-binding sites in the mRNA and amino acids near the ILARLN motif in the HAM1, HAM2, and HAM3 orthologous proteins were variable. Therefore, conservation of the miR171a-binding sites is more important for the regulation of proteins than that of the adjacent nucleotides in the mRNA and the corresponding amino acids in the proteins. The amino acids located upstream and downstream of the ILARLN motif are variable.

## 4. Discussion

In the study of miRNAs, there are many challenges. It is important to identify the targets for a particular miRNA and to determine the degree of miRNA binding to its target. The results from previous studies are contradictory. In particular, it has been suggested that the miRNA binds preferentially to the 3′UTR of the mRNA and that the binding is determined by the “seed” at the 5′-end of the miRNA [23–25]. However, miRNAs were shown to bind to the 5′UTR and CDS [26–28]. Recently, these results were further supported by a number of publications [14–17, 29]. It was shown that approximately 70% and 80% of miRNA-binding sites are located in the 5′UTR and CDS of mRNAs in animal and plant genes, respectively. Our results show that miRNA-binding sites can be located in the CDS of mRNAs and that the sites are highly conserved in the evolution of higher plants. There may be a reason for the localization of miRNA-binding sites in the CDS. These sites will be more conserved than those in the variable 5′UTR and 3′UTR mRNA regions. Localization of the binding sites in the CDS contributes to earlier miRNA-binding than localization in 3′UTR during the posttranscriptional regulation of gene expression. Our results reveal that such links could have been established a long time ago and are highly stable. The results were obtained with a high probability prediction of the miRNA-binding sites in the mRNAs of *SPL* and *HAM* genes. The occurrence of these interactions and their conservation in many plants show the fundamental role of gene expression regulation by miRNAs. The degree of miRNA binding to the mRNA is an indicator of the regulatory role of the miRNA in gene expression.

The absence of binding sites for a miRNA in mRNAs of some paralogs suggests that there is a way to protect part of the gene family expression from the effect of that miRNA. For example, about half of the *SPL* family genes are targets for the miR156 family. In animal cells, only the *PTPN12* gene out of the 16 *PTPN* paralogs strongly binds to has-miR-1279. Moreover, this relationship was highly conserved during evolution (unpublished data). Among the eight genes of the *MSH* family, only the mRNA of the *MSH6* gene has the binding site for has-miR-1279, and this binding site is preserved in the orthologs of the *MSH6 *gene. Such selective binding of an individual miRNA with mRNAs of other families of protein-coding genes is possible (unpublished data).

In this paper, we show that miRNA-binding sites with identical nucleotide sequences may be located in the CDS and 3′UTR of various genes, for example, *SPL3* and *SPL5* orthologs. However, a binding site in the 3′UTR is less conserved than that in the CDS. A similar result was obtained earlier with the *ZNF* gene family in animal cells [16].

Analysis of interaction schemes of the miRNA with the mRNA in Tables 3 and 4 shows that there is no clear preference for a particular part of the miRNA in binding to the mRNA. These data show a high degree of conservation of miRNA nucleotide sequences during evolution [30, 31].

According to the miRBase database, ath-miR156a–j and ath-miR157a–d belong to different families. However, all of these miRNA families have a common binding site in the mRNAs of the *SPL* gene family. It is likely that ath-miR156a–j and ath-miR157a–d are members of the same family. Similar arguments support the conclusion that ath-miR170 and ath-miR171a–c should be combined into the same family. The previously mentioned results should be considered for the distribution of miRNA in different families.

## Supplementary Material

Supplemental table S1: Nucleotide variability of miR171 binding sites in mRNA of *HAM* orthologous genes. The nucleotide sequences of miR171 binding sites are highly conserved in the mRNAs of 37 *HAM* orthologous genes and encode GAUAUUGGCGCGGCUCAAUCA sequence.
Supplemental Table S2: Amino acid variability of *HAM* orthologous proteins in regions with the ILARLN oligopeptide. The amino acid sequences of miR171-binding sites in *HAM* orthologous proteins are conserved and encode the same ILARLN motif.

## Figures and Tables

**Table 1:** Nucleotide variability of miR156a binding sites in mRNA of *SPL* paralogous genes and amino acid variability of SPL paralogous proteins in regions with the ALSLLS oligopeptide in *A. thaliana*.

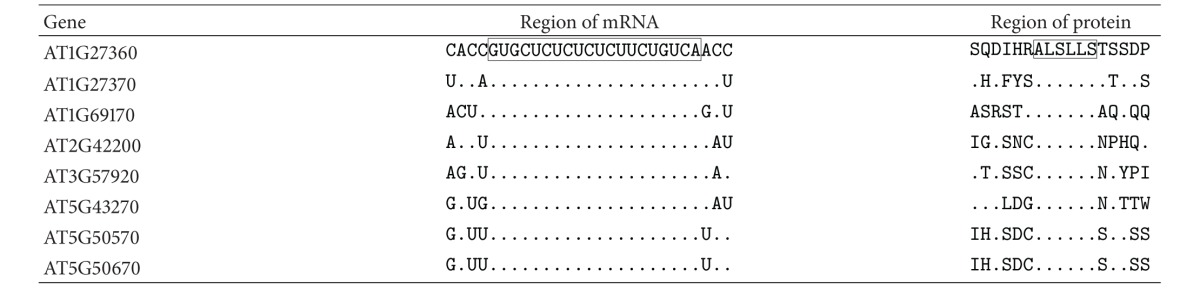

The conservative sequence is set in box.

**Table 2:** Characteristics of miR156a–j and miR157a–d binding sites in CDSs of *SPL* paralogous genes in *A. thaliana*.

Gene	Position in CDS, nt	Δ*G*/Δ*G*_*m*_, %						
		miR156a–f	miR156g	miR156h	miR156i	miR156j	miR157a–c	miR157d
AT1G27360	1211	91.4	88.6	93.9	99.3	100	91.7	92.5
AT1G27370	2365	91.1	88.4	93.6	99.3	100	91.4	92.5
AT1G69170	1295	91.4	88.6	93.9	99.3	100	91.0	92.5
AT2G42200	936	90.7	87.9	93.1	99.3	100	91.7	91.7
AT3G57920	844	90.7	87.9	93.1	99.3	100	91.7	91.7
AT5G43270	1186	90.7	87.9	93.1	99.3	100	91.7	91.7
AT5G50570	1100	90.2	87.9	92.6	98.8	100	89.5	91.2

**Table 3:** Schemes of miR156a–j and miR157a–d binding sites in the CDSs of *SPL* paralogous genes in *A. thaliana. *

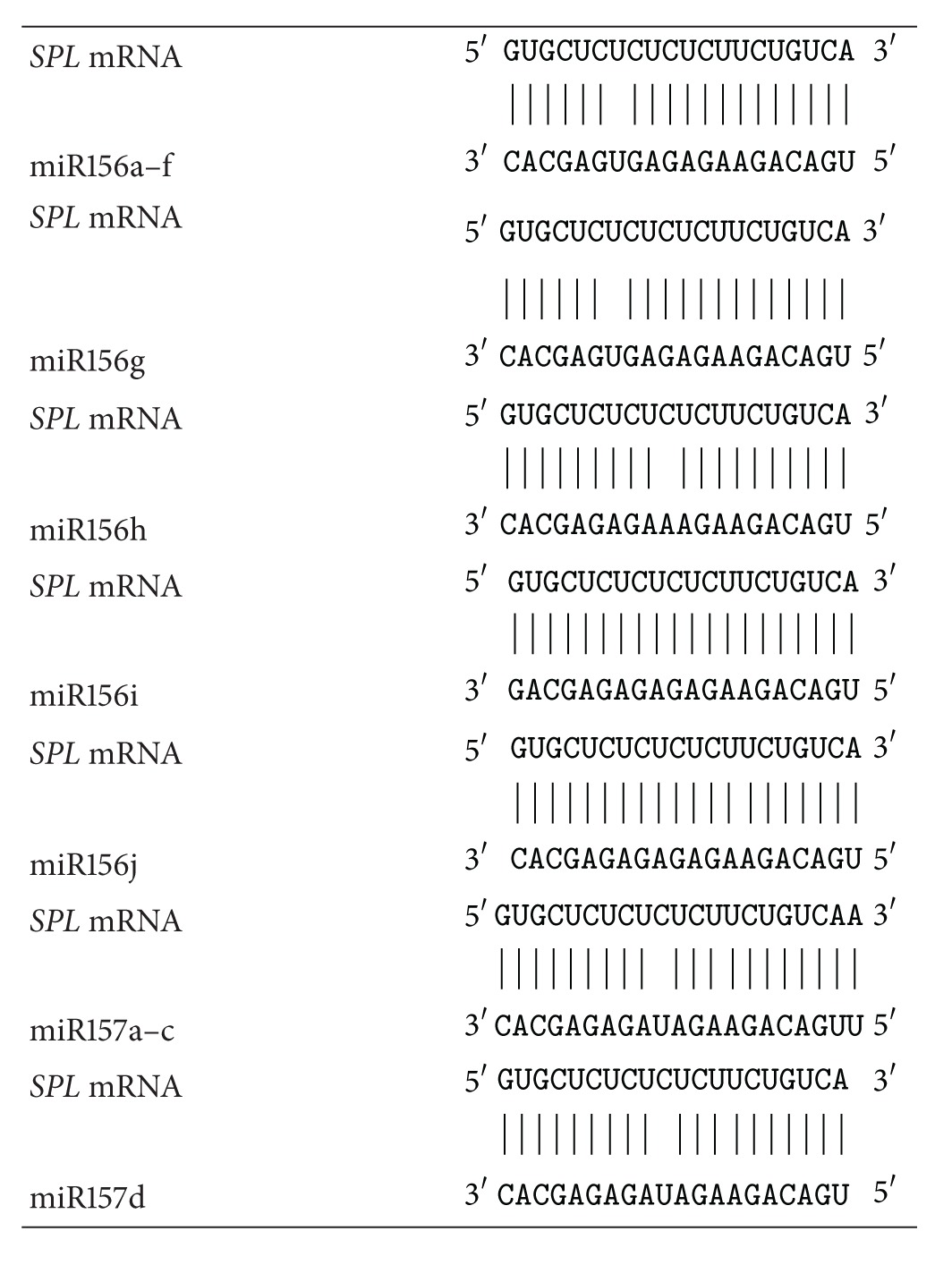

**Table 4:** Nucleotide variability of miR171a binding sites in mRNA of *HAM* paralogous genes and amino acid variability of HAM paralogous proteins in regions with the ILARLN oligopeptide in *A. thaliana*.

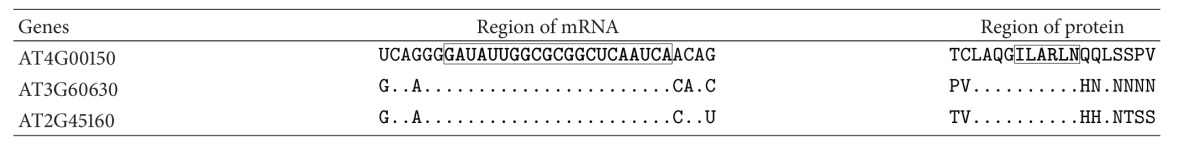

The conservative sequence is set in box.

**Table 5:** Characteristics of miR171a–c and ath-miR170 binding sites in mRNAs of *HAM* paralogous genes in *A. thaliana*.

Gene	Position in CDS, nt	Δ*G*/Δ*G*_*m*_, %		
		miR171a	miR171b,c	miR170
AT2G45160	884	100	86.9	98.8
AT3G60630	803	100	86.9	98.8
AT4G00150	674	100	86.9	99.5

**Table 6:** Schemes of miR171a–c and miR170 binding sites in CDSs of *HAM* paralogous genes in *A. thaliana*.

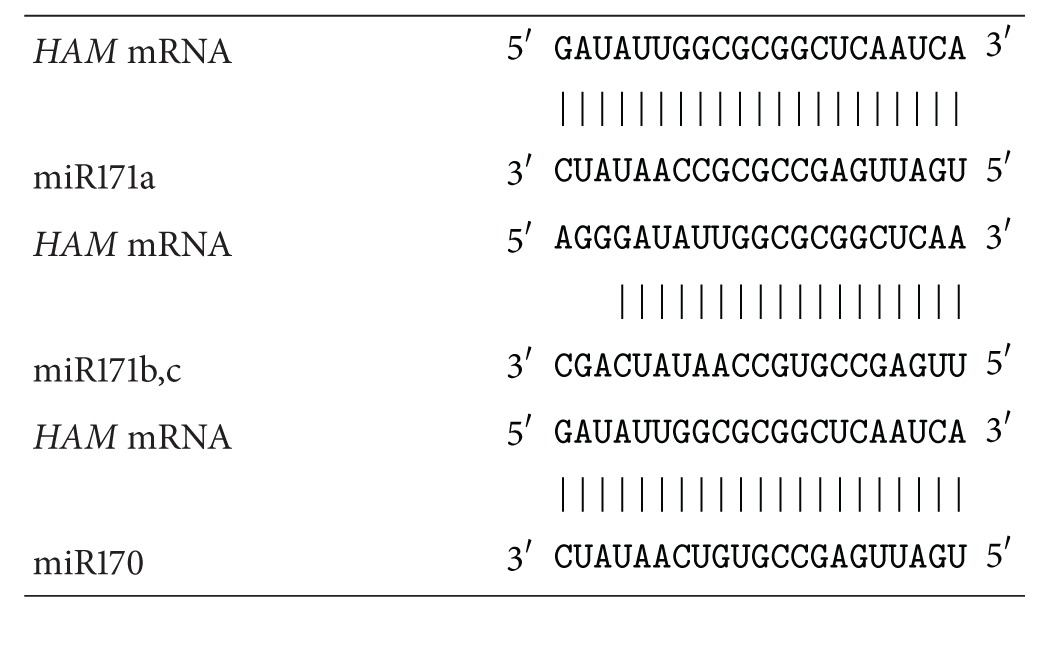

## References

[1] Cuperus JT, Fahlgren N, Carrington JC (2011). Evolution and functional diversification of *MIRNA* genes. *Plant Cell*.

[2] Tang Z, Zhang L, Xu C (2012). Uncovering small RNA-mediated responses to cold stress in a wheat thermosensitive genic male-sterile line by deep sequencing. *Plant Physiology*.

[3] Wu G, Poethig RS (2006). Temporal regulation of shoot development in *Arabidopsis thaliana* by miRr156 and its target SPL3. *Development*.

[4] Yamaguchi A, Wu M-F, Yang L, Wu G, Poethig RS, Wagner D (2009). The MicroRNA-regulated SBP-Box transcription factor SPL3 is a direct upstream activator of *LEAFY, FRUITFULL, and APETALA1*. *Developmental Cell*.

[5] Schwarz S, Grande AV, Bujdoso N, Saedler H, Huijser P (2008). The microRNA regulated SBP-box genes SPL9 and SPL15 control shoot maturation in Arabidopsis. *Plant Molecular Biology*.

[6] Cho SH, Coruh C, Axtella MJ (2012). miR156 and miR390 regulate tasiRNA accumulation and developmental timing in *Physcomitrella patens*. *Plant Cell*.

[7] Chorostecki U, Crosa VA, Lodeyro AF (2012). Identification of new microRNA-regulated genes by conserved targeting in plant species. *Nucleic Acids Research*.

[8] Meng Y, Shao C, Wang H, Chen M (2011). The regulatory activities of plant microRNAs: a more dynamic perspective. *Plant Physiology*.

[9] Hewezi T, Maier TR, Nettleton D, Baum TJ (2012). The arabidopsis microrna396-GRF1/GRF3 regulatory module acts as a developmental regulator in the reprogramming of root cells during cyst nematode infection. *Plant Physiology*.

[10] Kim JJ, Lee JH, Kim W, Jung HS, Huijser P, Ahn JH (2012). The microrNA156-SQUAMOSA promoter binding protein-like3 module regulates ambient temperature-responsive flowering via flowering locus in Arabidopsis. *Plant Physiology*.

[11] John B, Enright AJ, Aravin A, Tuschl T, Sander C, Marks DS (2004). Human microRNA targets. *Plos Biology*.

[12] Kiriakidou M, Nelson PT, Kouranov A (2004). A combined computational-experimental approach predicts human microRNA targets. *Genes and Development*.

[13] Krek A, Grün D, Poy MN (2005). Combinatorial microRNA target predictions. *Nature Genetics*.

[14] Grey F, Tirabassi R, Meyers H (2010). A viral microRNA down-regulates multiple cell cycle genes through mRNA 5’UTRs. *Plos pathogens*.

[15] Moretti F, Thermann R, Hentze MW (2010). Mechanism of translational regulation by miR-2 from sites in the 5′ untranslated region or the open reading frame. *RNA*.

[16] Schnall-Levin M, Rissland OS, Johnston WK, Perrimon N, Bartel DP, Berger B (2011). Unusually effective microRNA targeting within repeat-rich coding regions of mammalian mRNAs. *Genome Research*.

[17] Wang Y, Itaya A, Zhong X (2011). Function and evolution of a microRNA that regulates a caspi^2+^ -ATPase and triggers the formation of phased small interfering rnas in tomato reproductive Growth. *Plant Cell*.

[18] da Sacco L, Masotti A (2013). Recent insights and novel bioinformatics tools to understand the role of microRNAs binding to 5′ untranslated region. *International Journal of Molecular Sciences*.

[19] Wu G, Park MY, Conway SR, Wang J-W, Weigel D, Poethig RS (2009). The sequential action of miR156 and miR172 regulates developmental timing in arabidopsis. *Cell*.

[20] Engstrom EM, Andersen CM, Gumulak-Smith J (2011). Arabidopsis homologs of the petunia HAIRY MERISTEM gene are required for maintenance of shoot and root indeterminacy. *Plant Physiology*.

[21] Krüger J, Rehmsmeier M (2006). RNAhybrid: MicroRNA target prediction easy, fast and flexible. *Nucleic Acids Research*.

[22] Crooks GE, Hon G, Chandonia J-M, Brenner SE (2004). WebLogo: a sequence logo generator. *Genome Research*.

[23] Lim LP, Lau NC, Garrett-Engele P (2005). Microarray analysis shows that some microRNAs downregulate large numbers of-target mRNAs. *Nature*.

[24] Farh KK-H, Grimson A, Jan C (2005). Biochemistry: the widespread impact of mammalian microRNAs on mRNA repression and evolution. *Science*.

[25] Lewis BP, Burge CB, Bartel DP (2005). Conserved seed pairing, often flanked by adenosines, indicates that thousands of human genes are microRNA targets. *Cell*.

[26] Ørom UA, Nielsen FC, Lund AH (2008). MicroRNA-10a binds the 5′UTR of ribosomal protein mRNAs and enhances their translation. *Molecular Cell*.

[27] Lee I, Ajay SS, Jong IY (2009). New class of microRNA targets containing simultaneous 5′-UTR and 3′-UTR interaction sites. *Genome Research*.

[28] Zhou X, Duan X, Qian J, Li F (2009). Abundant conserved microRNA target sites in the 5′-untranslated region and coding sequence. *Genetica*.

[29] Issabekova AS, Berillo OA, Regnier M, Ivashchenko AT (2012). Interactions of intergenic microRNAs with mRNAs of genes involved in carcinogenesis. *Bioinformation*.

[30] Miura S, Nozawa M, Nei M (2011). Evolutionary changes of the target sites of two MicroRNAs encoded in the Hox gene cluster of Drosophila and other insect species. *Genome Biology and Evolution*.

[31] Nozawa M, Miura S, Nei M (2010). Origins and evolution of MicroRNA genes in plant species. *Genome Biology and Evolution*.

