# Supplementary material for: miR156- and miR171-Binding Sites in the Protein-Coding Sequences of Several Plant Genes
Source: Biomed Res Int. 2013 Jul 11;2013:307145. doi: 10.1155/2013/307145 (PMC3727082; doi:10.1155/2013/307145)
Supplement: Supplementary file 1 — Supplemental table S1: Nucleotide variability of miR171 binding sites in mRNA of HAM orthologous genes. The nucleotide sequences of miR171 binding sites are highly conserved in the mRNAs of 37 HAM orthologous genes and encode GAUAUUGGCGCGGCUCAAUCA sequence. Supplemental Table S2: Amino acid variability of HAM orthologous proteins in regions with the ILARLN oligopeptide. The amino acid sequences of miR171-binding sites in HAM orthologous proteins are conserved and encode the same ILARLN motif. [file 307145.f1.pdf]

Supplemental table S1: Nucleotide variability of miR171 binding sites in mRNA of HAM orthologous genes

Organism	Gene accession	Region of CDS in mRNA of HAM orthologs (the conservative sequence is set in bold)
A. thaliana	AT2G45160	GCAAGG**GAUAUUGGCGCGGCUCAAUCA**CCAUCU
A. thaliana	AT3G60630	GCAAGG**GAUAUUGGCGCGGCUCAAUCA**CAACCU
A. thaliana	AT4G00150	UCAGGG**GAUAUUGGCGCGGCUCAAUCA**ACAGCU
A. lyrata	ARALYDRAFT_483651	GCAAGG**GAUAUUGGCGCGGCUCAAUCA**CCAUCU
A. lyrata	ARALYDRAFT_486537	GCAAGG**GAUAUUGGCGCGGCUCAAUCA**CAACCU
A. lyrata	ARALYDRAFT_490555	UCAGGG**GAUAUUGGCGCGGCUCAAUCA**ACAGCU
B. distachyon	LOC100822245	ACGCGA**GAUAUUGGCGCGGCUCAAUUA**UCGGCU
B. distachyon	LOC100828697	GCGAGA**GAUAUUGGCGCGGCUCAAUCA**CCAGCU
B. distachyon	LOC100834684	GCGCGA**GAUACUGGCACGGCUCAAUCA**UCGGCU
B. distachyon	LOC100841394	CAGGGA**GAUAUUGGCGCGGCUCAAUCA**CCAUGG
G. max	LOC100792973	GCAUGG**GAUAUUGGCGCGGCUCAAUCA**CCAACU
G. max	LOC100801785	GCAAGG**GAUAUUGGCGCGGCUCAAUCA**CCAGCU
G. max	LOC100805945	GCACGG**GAUAUUGGCGCGGCUCAAUCA**CCAACU
M. truncatula	MTR_4g026490	GCAAGG**GAUAUUGGCGCGGCUCAAUCA**CCAGCU
O. sativa	Os02g0662700	GCGAGA**GAUAUUGGCGCGGCUCAAUCA**ACAGCU
O. sativa	Os02g0663100	GCGCGA**GAUAUUGGCGCGGCUCAAUCA**ACAGCU
O. sativa	Os04g0555000	GCGAGA**GAUAUUGGCGCGGCUCAAUCA**GCAGCU
O. sativa	Os10g0551200	CCGCGA**GAUAUUGGCGCGGCUCAAUUA**CCGGCU
P. patens	PHYPADRAFT_143602	UAAAGC**GAUAUUGGCGCGGCUCAAUCA**GCACAU
P. trichocarpa	POPTR_409708	GCAAGG**GAUAUUGGCGCGGCUCAAUCA**CCAGCU
P. trichocarpa	POPTR_591663	GCAAGG**GAUAUUGGCGCGGCUCAAUCA**CCAGCU
P. trichocarpa	POPTR_784409	GCAAGG**GAUAUUGGCGCGGCUCAAUCA**ACAGCU
P. trichocarpa	POPTRDRAFT_409708	GCAAGG**GAUAUUGGCGCGGCUCAAUCA**CCAGCU
R. communis	RCOM_1062060	GCAAGG**GAUAUUGGCGCGGCUCAAUCA**ACAGCU
R. communis	RCOM_1610560	GCAAGG**GAUAUUGGCGCGGCUCAAUCA**CCAGCU
S. bicolor	SORBIDRAFT_01g029650	CCGCGA**GAUAUUGGCGCGGCUCAAUUA**UCGGCU
S. bicolor	SORBIDRAFT_01g050333	CCGCGA**GAUACUGGCGCGGCUCAACUA**CCTGCU
S. bicolor	SORBIDRAFT_04g032570	GCGAGA**GAUAUUGGCGCGGCUCAAUCA**CCAGCU
S. bicolor	SORBIDRAFT_04g032590	GCGAGA**GAUAUUGGCGCGGCUCAAUCA**CCAGCU
S. bicolor	SORBIDRAFT_06g024820	GCUCGA**GAUAUUGGCGCGGCUCAAUCA**GCAGCU
S. moellendorffii	SELMODRAFT_453332	UCAGUC**GAUAUUGGCGCGGCUCAAUCA**GCAUUU
V. vinifera	LOC100267664	GCAAGG**GAUAUUGGCGCGGCUCAAUCA**CCAGCU
Z. mays	LOC100272800	GCGCGA**GAUAUUGGCGCGGCUCAAUCA**CCGGCU
Z. mays	LOC100279263	GCGAGA**GAUAUUGGCGCGGCUCAAUCA**CCAGCU
Z. mays	LOC100279420	GCGAGC**GAUAUUGGCGCGGCUCAAUCA**CCAGCU
Z. mays	LOC100381701	GCGCGA**GAUAUUGGCGCGGCUCAAUCA**GCAGCU
Z. mays	LOC100382187	GCGAGA**GAUAUUGGCGCGGCUCAAUCA**GCAGCU

Supplemental Table S2: Amino acid variability of HAM orthologous proteins in regions with the ILARLN oligopeptide

Organism	Gene accession	Region of HAM orthologous protein (the conservative sequence is set in bold)
A. thaliana	AT2G45160	TVLAQG**ILARLN**HHLNTS
A. thaliana	AT3G60630	PVLAQG**ILARLN**HNLNNN
A. thaliana	AT4G00150	TCLAQG**ILARLN**QQLSSP
A. lyrata	ARALYDRAFT_483651	TVLAQG**ILARLN**HHLNTS
A. lyrata	ARALYDRAFT_486537	PVLAQG**ILARLN**HNLNNN
A. lyrata	ARALYDRAFT_490555	TCLAQG**ILARLN**QQLSSP
B. distachyon	LOC100822245	AHGARE**ILARLN**YRLPAA
B. distachyon	LOC100828697	STGARE**ILARLN**HQLPPV
B. distachyon	LOC100834684	GFGARE**ILARLN**HRLPAA
B. distachyon	LOC100841394	STGARE**ILARLN**HHGLLP
G. max	LOC100792973	LELAHG**ILARLN**HQLSPI
G. max	LOC100801785	PVHAQG**ILARLN**HQLSPI
G. max	LOC100805945	LELAHG**ILARLN**HQLSPI
M. truncatula	MTR_4g026490	PVQAQG**ILARLN**HQLSPI
O. sativa	Os02g0662700	SVGARE**ILARLN**QQLPQL
O. sativa	Os02g0663100	SVGARE**ILARLN**QQLPPL
O. sativa	Os04g0555000	SIGARE**ILARLN**QQLPPI
O. sativa	Os10g0551200	AFGARE**ILARLN**YRLPAA
P. trichocarpa	POPTR_409708	PVLAQG**ILARLN**HQLSVP
P. trichocarpa	POPTR_591663	PVLAQG**ILARLN**HQLSLP
P. trichocarpa	POPTR_784409	FLHAQG**ILARLN**QQLSPT
P. trichocarpa	POPTRDRAFT_409708	PVLAQG**ILARLN**HQLSVP
P. patens	PHYPADRAFT_143602	AEMAKA**ILARLN**QHISPS
R. communis	RCOM_1062060	FSHAQG**ILARLN**QQLSPI
R. communis	RCOM_1610560	PALAQG**ILARLN**HQLSLS
S. bicolor	SORBIDRAFT_01g029650	AFGARE**ILARLN**YRLPAV
S. bicolor	SORBIDRAFT_01g050333	VLGARE**ILARLN**YLLPPA
S. bicolor	SORBIDRAFT_04g032570	SIGARE**ILARLN**HQLPPL
S. bicolor	SORBIDRAFT_04g032590	SIGARE**ILARLN**HQLPPL
S. bicolor	SORBIDRAFT_06g024820	SIGALE**ILARLN**QQLPPV
S. moellendorffii	SELMODRAFT_453332	NHVAQS**ILARLN**QHLFCH
V. vinifera	LOC100267664	TILAQG**ILARLN**HQLSPI
Z. mays	LOC100272800	STGARE**ILARLN**HRLPSP
Z. mays	LOC100279263	SIGARE**ILARLN**HQLPLL
Z. mays	LOC100279420	SIGARA**ILARLN**HQLPPL
Z. mays	LOC100381701	SIGGRE**ILARLN**QQLPPI
Z. mays	LOC100382187	SIGARE**ILARLN**QQLPPI
